# P-794. An eight-year experience of active case-finding for tuberculosis cases using mass chest radiography among prisoners in Songkhla Province, Thailand

**DOI:** 10.1093/ofid/ofae631.986

**Published:** 2025-01-29

**Authors:** Sujinda Ruangchan, Pornthip Saengsanga, Nongnuch Kiamkan

**Affiliations:** Songkhla Provincial Hospital, Songkhla Province, Songkhla, Thailand; Songkhla Hospital, Mueang, Songkhla, Thailand; Songkhla Provincial Hospital, Songkhla Province, Songkhla, Thailand

## Abstract

**Background:**

Thailand is classified by the World Health Organization (WHO) as a high tuberculosis-burden country with an expected that there are 120,000 TB incidences per year in 2016. Reported cases of tuberculosis among prisoners may account for 30% of tuberculosis with an incidence of 880-5,803 cases per 100,000 inmates in low- to middle-income countries. Beginning in 2017, an active case–finding strategy for tuberculosis was implemented in all prisons in Thailand. Mass chest radiograph screening is performed on all prisoners to identify and bring into treatment tuberculosis cases as soon as possible.

Incidence of pulmonary tuberculosis among prisoners in Songkhla Province in the South of Thailand 2016-2023

**Figure:**
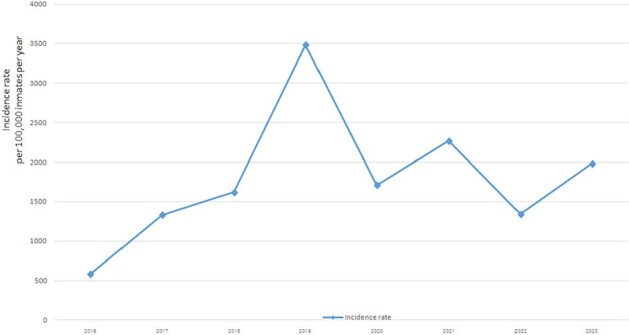

**Methods:**

A retrospective descriptive study was conducted at Songkhla Provincial Hospital between 2016 and 2023. Inmates from four prisons in Songkhla Province were assessed by tuberculosis screening using mass chest radiographs yearly from 2017. If abnormal chest radiography revealed compatibility with tuberculosis, sputum microscopy and sputum GeneXpert were performed. Data were analyzed using descriptive statistics

**Results:**

After implementing the active case-finding strategy in 2017, the incidence of pulmonary tuberculosis in prisons rose from 584 cases per 100,000 inmates in 2016 to 3,483 per 100,000 inmates in 2019. Then the trend declined to 2,264 per 100,000 inmates in 2021 and 1,982 per 100,000 inmates in 2023. Tuberculosis cases in the prisons accounted for 15% in 2016 and continued to rise to 60% in 2019 then decline to 41% in 2023 of all tuberculosis cases in Songkhla Province. Furthermore, sputum microscopy or GeneXpert detected tuberculosis positive between 10% and 16% of all tuberculosis cases in the four prisons. The rates of treatment success were 90%-94% and the case fatality rates were 11-58 cases per 100,000 inmates at the four prisons.

**Conclusion:**

The active case-finding strategy using mass chest radiography increases the early detection of tuberculosis cases and early treatment in prisons. After 3 years of implementing strategies, the incidence of pulmonary tuberculosis has declined

**Disclosures:**

**All Authors**: No reported disclosures

